# Consistency in mutualism relies on local, rather than wider community biodiversity

**DOI:** 10.1038/s41598-020-78318-x

**Published:** 2020-12-04

**Authors:** Katie Dunkley, Jo Cable, Sarah E. Perkins

**Affiliations:** 1 School of Biosciences, Cardiff University, Cardiff, CF10 3AX UK; 2 Department of Zoology, University of Cambridge, Cambridge, CB2 3EJ UK

**Keywords:** Animal behaviour, Behavioural ecology, Community ecology, Ecosystem ecology, Ecosystem services, Conservation biology

## Abstract

Mutualistic interactions play a major role in shaping the Earth’s biodiversity, yet the consistent drivers governing these beneficial interactions are unknown. Using a long-term (8 year, including > 256 h behavioural observations) dataset of the interaction patterns of a service-resource mutualism (the cleaner-client interaction), we identified consistent and dynamic predictors of mutualistic outcomes. We showed that cleaning was consistently more frequent when the presence of third-party species and client partner abundance locally increased (creating choice options), whilst partner identity regulated client behaviours. Eight of our 12 predictors of cleaner and client behaviour played a dynamic role in predicting both the quality (duration) and quantity (frequency) of interactions, and we suggest that the environmental context acting on these predictors at a specific time point will indirectly regulate their role in cleaner-client interaction patterns: context-dependency can hence regulate mutualisms both directly and indirectly. Together our study highlights that consistency in cleaner-client mutualisms relies strongly on the local, rather than wider community—with biodiversity loss threatening all environments this presents a worrying future for the pervasiveness of mutualisms.

## Introduction

Nearly every organism on the planet is directly or indirectly engaged in some form of mutualism^1^. Such interactions, which involve cooperation between species, are core drivers in shaping communities and have played a central role in ecological and evolutionary processes^2^. Despite the importance of mutualisms, we still do not understand what creates the spatial and temporal^3,4^ variations in interaction outcomes that are so frequently observed. This heterogeneity has led to the hypothesis that mutualisms are context-dependent^5–7^, but our knowledge concerning the biotic and abiotic contexts^6^ that favour the evolution and maintenance of mutualistic interactions is limited. As a result, it is unclear how mutualistic patterns and environmental variables interlink to shape ecological communities^8^. With large environmental shifts threatening most ecosystems^9^, it is vital that we understand the underlying dynamics of an interaction pervasive across the animal kingdom.

Most mutualisms are service-resource interactions^10^ where a beneficial act ‘the service’ (e.g. pollination^11^, parasite removal^12^ or myrmecophily^13^) is traded for a food resource (e.g. nectar, ectoparasites or honeydew). At their simplest level, mutualisms involve one individual interacting with another, but over time, mutualists can interact with a large number of species^14^. Partner choice is thus a driving force behind mutualism evolution^15^ since a strong preference for one partner screens out others^16^. Many studies of mutualism dynamics focus on plant-pollinator interactions^2,17^, in which the pollinator selects the static, non-mobile plant. In more complex interactions however, where both interacting species are motile, both partners can make choices of whom to interact with and how^18^. Perhaps, the best-known example is the cleaner-client interactions: a cleaner removes ectoparasites and debris from the body of another other species (known as clients^19^), and on one reef alone multiple species can act as cleaners and interact with a large proportion of the reef fish^14,20,21^. As a result, cleaning can be considered a central interaction for reef communities^14^. Cleaners may occupy coral head cleaning stations^22^ and clients can solicit cleaning at these stations by presenting their body to cleaners (termed posing^19^). This behaviour however does not always guarantee cleaning, and for some clients, they do not have to pose at all to be cleaned^12,23^. Thus, both individual clients and cleaners can select who to interact with, and these choices and subsequent interactions, can influence future interactions^18,24^. It is currently unknown however whether the same contextual factors that favour one partner’s choice (e.g. the cleaner) are just as important for the other (e.g. the client)^5^.

The widespread maintenance of mutualisms is an evolutionary conundrum since the relationship is founded upon a conflict of interest. Mutualisms can be best thought of the reciprocal exploitation of resources/services of partners^25^ whereby the extent of the cost experienced by one partner mirrors the benefit obtained by the other. As a result, both partners will try to maximise their own benefits with the least possible investment^26^. The mechanisms behind this can be considered in the context of ‘biological market theory’^15^. Partners differ in the quality^12,27–29^ and quantity^29–31^ of material they host or trade depending on their traits, and the relative value of each partner to the other will depend upon their abundance and the presence of other third-parties within the environment (i.e., those in the community that are external, but available, to the focal mutualism at a specific time point^32^). A decrease in the abundance of one partner, for example, could be detrimental to the other, or facilitate a shift to a different partner^21,33,34^. In cleaning interactions for example, the quality and quantity of the cleaning service provided can vary with the clients’ identity and behaviour^12,18,24^, the cleaners identity (across^14,21,35^ and within-species^18,36^) and the number and distribution of cleaners and or clients present in an environment^21,37–39^, whilst the quality and quantity of the clients’ resources can vary between species (individual client species differ in the nutritional content that they represent to cleaners^27,29,30,40^) and with the clients’ propensity to visit stations and engage in cleaning interactions^23,24^. Ultimately changes in partner diversity will significantly re-wire mutualistic patterns and networks^41–43^, directly or indirectly harming or benefiting the participating species^43,44^. The combined effect of three key contextual factors: partner identity, partner abundance and the presence of third-party species, could thus impact mutualistic outcomes. What is not currently clear is how partner diversity alters mutualistic patterns tempo-spatially and how these patterns shift along species diversity gradients^5,16^.

Here we aimed to quantify how context-dependency governs cleaner-client interactions by identifying consistent and dynamic predictors of both cleaner and client behaviour. Using 8 years of behavioural observations on the same coral reef and across the same cleaning stations (256 h and 30 min of observations across 82 cleaning stations), we first quantified the variability in cleaner and client behaviour (cleaning and posing) over time and space (within and across years). Secondly, we identified which contextual factors, relating to partner identity, partner abundance and the presence of third-party species, are the most important and consistent predictors of cleaner and client behaviour. Both the quantity and quality of the behaviours were considered by quantifying cleaning and posing frequencies and durations. Ultimately, we aimed to identify whether partner identity, partner abundance or the presence of third-party species is the most important for maintaining the occurrence of an iconic, and central mutualism.

## Results

### How do cleaner-client interactions vary tempo-spatially?

Temporally, cleaning frequency and client posing behaviours differed between 8 years in the same location (GLMM, clean frequency: χ^2^_1_ = 34.42, *p* < 0.001, pose frequency: χ^2^_1_ = 78.51, *p* < 0.001, clean duration: χ^2^_1_ = 11.17, *p* = 0.132, pose duration: χ^2^_1_ = 22.89, *p* = 0.002, Tukey’s *p* < 0.05, see Supplementary Table 1 and Supplementary Fig. 1). Neither cleaning frequency nor duration differed with time of day, a pattern that was observed across all 8 years (GLMM, clean frequencies and durations all *p* > 0.05, Supplementary Table 1). Clients posed more frequently earlier in the day, in only 3 out of 8 years (pose frequency GLMM, 2010: z = − 2.31, *p* = 0.021, 2011: z = − 3.58, *p* < 0.001, 2013: z = − 3.43, *p* < 0.001, pose durations GLMM, *p* > 0.05, Supplementary Table 1).

**Figure 1:**
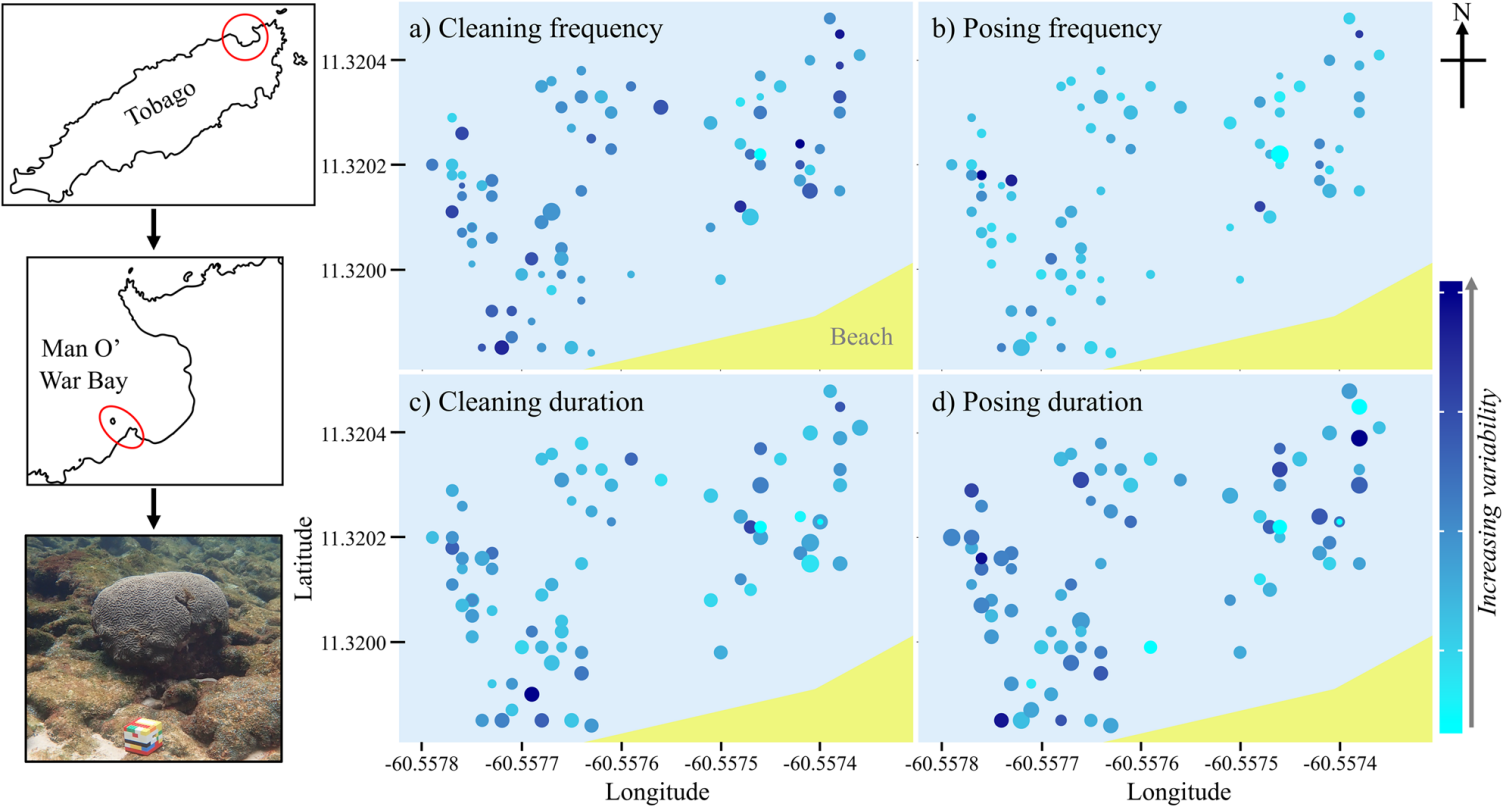
Random spatial patterning of cleaner-client interactions. Data were collected on cleaning interactions of sharknose goby (*Elacatinus evelynae*) and their clients on Booby Reef, Man O’ War Bay Tobago. Each circle represents a cleaning station where interactions repeatedly took place and the scaled size of the circle represents mean (**a**) cleaning frequencies, (**b**) posing frequencies, (**c**) cleaning durations and (**d**) posing durations, with larger circles showing increased frequencies or durations across years (predicted values from GLMMs). The colour of each circle represents the variation of this mean value and is based on the relative standard error (RSE). The RSE (expressed as a %) is similar to the coefficient of variation but provides a measure of variability whilst accounting for the mean and variable sample sizes for each location. Photograph (credit: Katie Dunkley) shows example of an isolated (no other neighbouring) cleaning station. Maps (**a**–**d**) were created as scatterplots using GPS fixes (collected by Katie Dunkley in 2018) for each individual cleaning station and the beach edge. Tobago maps were drawn by Katie Dunkley using ‘Graphic for iPad’ (version 3.5.2).

Spatially, cleaning frequency and duration differed between cleaning stations across 8 years, as did the frequency of client posing (Fig. 1, LRT, clean frequency: χ^2^_1_ = 22.65, *p* < 0.001, clean duration: χ^2^_1_ = 25.09, *p* < 0.001, pose frequency: χ^2^_1_ = 23.65, *p* < 0.001, pose duration: χ^2^_1_ = 0.37, *p* = 0.543). There was no evidence however that cleaner-client interaction outcomes showed spatial autocorrelation (Fig. 1, Mantel’s tests all *p* > 0.100, Supplementary Table 1). Similarly, cleaning stations that were situated close to one another (i.e. considered aggregated) did not differ in their cleaner-client interaction patterns across years compared to those cleaning stations that were considered more isolated (Fig. 1, Pearson correlation, clean and pose frequencies and durations with an ‘aggregation PC1 score’ all *p* > 0.100, Supplementary Table 1).

### Contextual factors predicting cleaning behaviour

**Figure 2:**
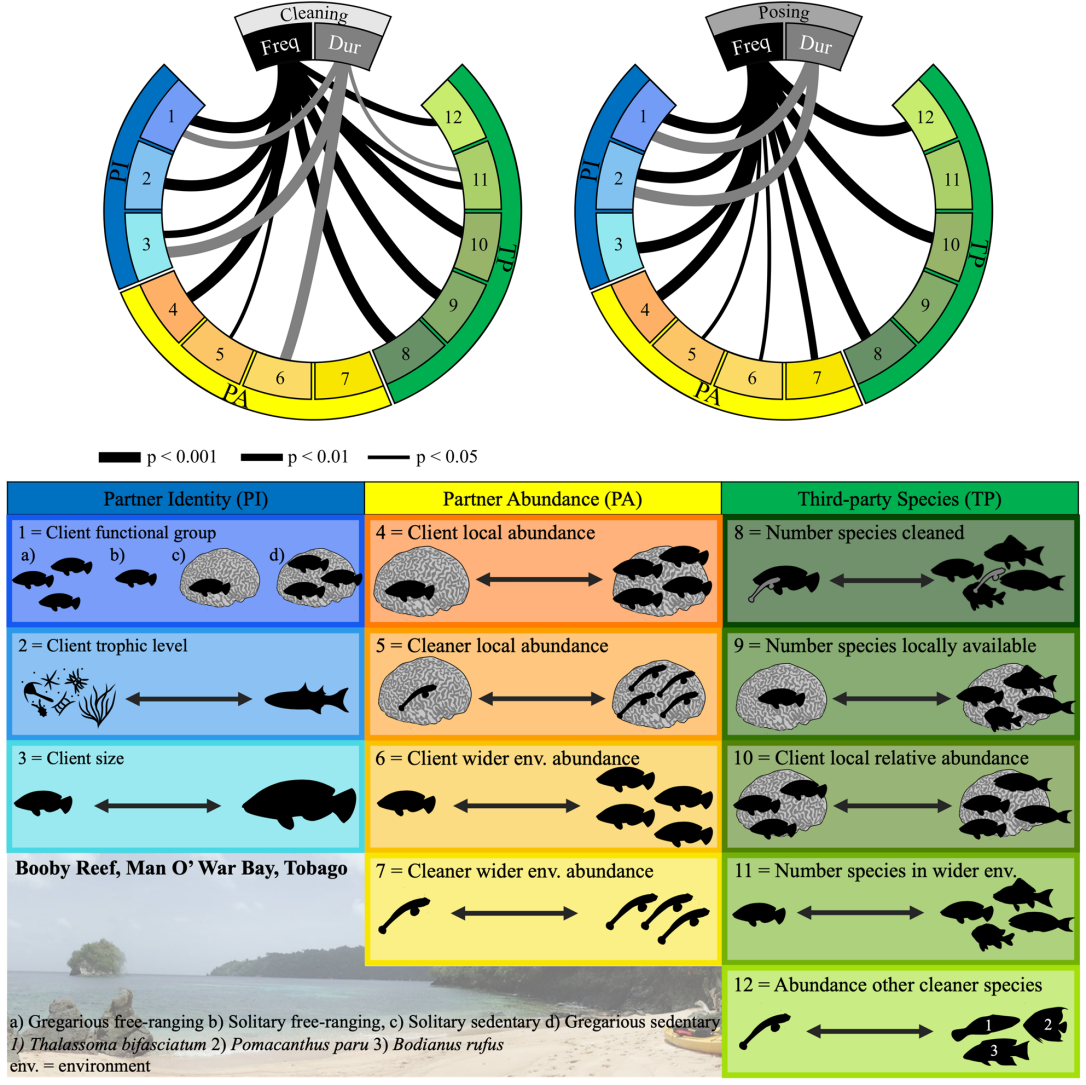
Twelve contextual factors, relating to partner identity (PI), partner abundance (PA) and the presence of third-party species (TP), driving cleaner-client interaction outcomes from a long-term 8-year empirical dataset. Lines show significant predictors of cleaning and posing frequency and duration with thickness indicating significance levels (for full test results see Supplementary Table 2). Predictors are numbered from 1 to 12 and are outlined in the table. For full details see Table 2 in methods. Photograph credit: Kathryn Whittey, vector graphics were created by Katie Dunkley using ‘Graphic for iPad’ (version 3.5.2).

**Figure 3:**
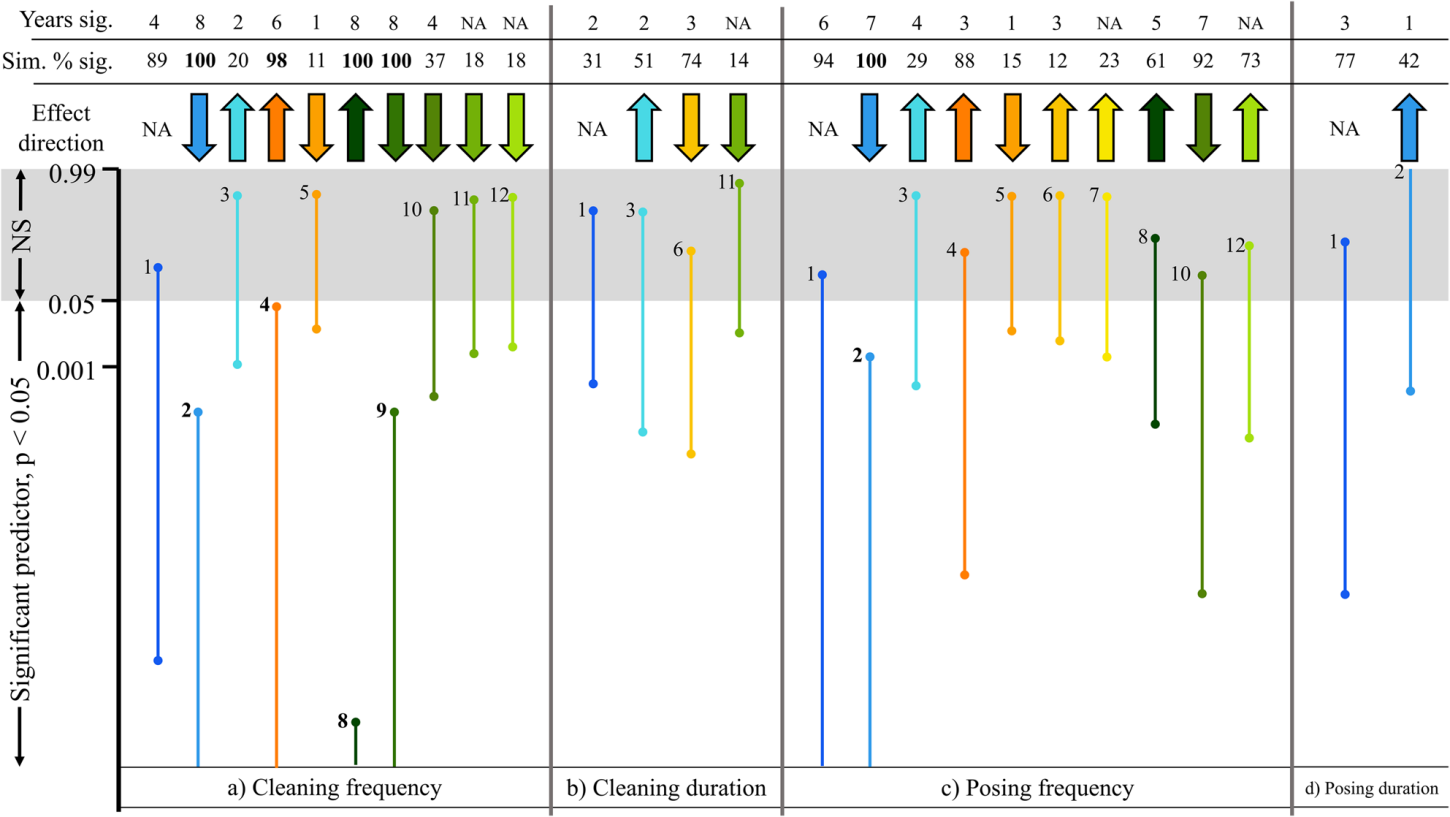
Consistent and dynamic contextual predictors of cleaning and client posing behaviour (frequencies and durations). From an 8 year dataset of 1539 observations, random subsamples were selected (n = 192 observations per simulation) and GLMM models were re-run 1000 times. Bar lengths show the range of generated p-values for each predictor across these simulated models, whilst ‘Sim. % sig.’ shows the percentage of times each predictor significantly predicted (*p* < 0.05) cleaner and/or client behaviour (cleaning/posing frequencies and durations) out of 1000. P-value ranges were plotted on a logit scale while the y axis values show the position of the untransformed p-values (NS = not significant, *p* > 0.05). The years significant (sig.) represents the number of years within our dataset (out of 8) the predictor was significant (*p* < 0.05) (see Supplementary Table 2) and the effect direction shows the positive or negative effect each predictor had on cleaner and client behaviour. Predictors are numbered from 1 to 12 (with colours matching Fig. 2) and bold formatting represents those factors which were consistent predictors of cleaner or client behaviour. Effect directions could not be obtained for the categorical factor, client functional group, and some contextual factors did not differ within years: these values are denoted by ‘NA’. 1 = client functional group 2 = client trophic level 3 = client body size 4 = client local abundance 5 = cleaner local abundance 6 = client wider environment abundance 7 = cleaner wider environment abundance 8 = number species cleaned 9 = number species locally available 10 = client local relative abundance 11 = number species in wider environment and 12 = abundance of other cleaner species.

Twelve contextual factors relating to partner abundance, partner identity and the presence of third-party species were identified (Fig. 2, predictors 1–12). Ten of these 12 factors significantly predicted cleaning frequency (Fig. 2, GLMM _adj_R^2^ = 39.2%). Factors relating to the presence of third-party species were together the most important predictor (Fig. 2, mean R^2^ proportion change per group when factor(s) added last to final model, third-party species = 7.4%, partner identity = 6.3%, partner abundance = 4.8%). The number of species cleaned was the most important single predictor having a positive effect on cleaning frequency (Fig. 3, mean R^2^ proportion change per group when factor added last to final model = 27%).

Although ten contextual factors predicted cleaning frequency only four of these were consistent predictors (Fig. 3a, proportion of 1000 simulations significant > 95%; see Supplementary Table 3a). Cleaning frequency consistently increased with the clients’ local abundance and the number of species cleaned but decreased when the clients’ trophic level and the number of species locally available was high (Fig. 3a). The remaining six significant, but ephemeral predictors can hence be considered dynamic predictors of cleaning frequency (Fig. 3a).

It was not possible to identify which of the four consistent contextual factors was the most important consistent predictor of cleaning frequency, since the identity of the most important predictor variable varied across each of the 1000 simulations. The clients’ local abundance, however, was never the most important consistent predictor of cleaning frequency (importance identified using ranked absolute standardised β values, 95% CI of β value across 1000 simulations: client trophic level [0.11, 0.26], number species cleaned [0.14, 0.25], number species locally available [0.10, 0.22] and client local abundance [0.04, 0.15]).

Only four of the 12 factors predicted cleaning duration (Fig. 2, GLMM _adj_R^2^ = 24.3%). Client wider abundance was the most important predictor, with decreased durations when abundances were high (Fig. 3b, R^2^ change = 5.1%): rarer clients were hence cleaned for longer. None of these factors, however, were consistent predictors of duration (Fig. 3b, proportion of 1000 simulations significant all *p* > 0.2). This result was also reflected within years, since factors only predicted cleaning duration in a maximum of 3 of the 8 years (Fig. 3b, Supplementary Table 3b). After accounting for the role of factors in predicting cleaner behaviour across years, cleaning stations still differed from one another in their cleaning durations (LRT, χ^2^_1_ = 15.67, *p* < 0.001).

### Contextual factors predicting client behaviour

The frequency of client posing was predicted by 10 factors, relating to partner abundance, partner identity and the presence of third-party species (Fig. 2, GLMM _adj_R^2^ = 33.1%). Posing durations were only predicted by two factors relating to partner identity (Fig. 2, GLMM _adj_R^2^ = 28.7%).

Contrasting cleaning, factors relating to the clients’ identity were the most important predictors of their posing frequencies and durations (Fig. 2, pose frequency: mean R^2^ proportion change per factor group, partner identity = 6.8%, partner abundance = 1.6%, third party species = 6.2%, posing duration: partner identity = 19.4%). The clients’ functional group was the most important predictor of both posing frequencies and durations across years (posing frequency: R^2^ change = 13.2%, posing duration: R^2^ change = 5.7%): solitary free-ranging clients posed more frequently and for longer than the other three types (Fig. 2). After accounting for the role of factors in predicting client behaviour across years, cleaning stations still differed from one another in their posing frequencies (LRT, χ^2^_1_ = 18.52, *p* < 0.001).

Posing frequencies were only consistently predicted by the clients’ trophic level (Fig. 3c, proportion of 1000 simulations significant = 100%). Frequencies consistently decrease with increased trophic level: predatory species posed less frequently. This result was also reflected in the within year analysis; trophic levels predicted posing frequencies in 7 out of 8 years (Fig. 3c, Supplementary Table 3c, GLMM, 2012 z = − 1.85, *p* = 0.065). Like cleaning durations, no factors across (1000 simulated GLMMs, proportion significant *p* > 0.100, Fig. 3d) or within years (Fig. 3d, Supplementary Table 3d) consistently predicted posing durations.

## Discussion

This long-term 8 year study on a cleaner-client mutualism has quantified which contextual factors govern interaction outcomes for both partners. Here, cleaner-client interaction patterns varied temporally and spatially, with cleaning (frequency and duration) and client posing frequencies differing across cleaning stations within the environment. Different drivers influenced the cleaners versus clients’ behaviour: cleaning was predominantly regulated by the presence of third-party species and partner abundance locally available (creating local choice options), whilst partner identity regulated client posing. Most identified predictors of cleaner and client behaviours played a dynamic role in predicting both the quality and quantities of interactions. This study demonstrates that the local, rather than wider environment plays a pivotal and consistent role in mutualism dynamics and highlights the need to consider multiple contextual factors when investigating mutualistic patterns.

Our time-series of data enabled us for the first time to confidently identify factors that are dynamic (as also inferred by contrasting results found across previous studies^35,45,46^) or consistent predictors of cleaner-client behaviour. Partner quality (defined as how valuable a partner is to another) has been previously considered a continuously varying trait depending on underlying genetic, phenotypic and/or spatial heterogeneity^47^. Indeed, different clients are asymmetric in the quality and quantity of the material they host (e.g. parasites^29,30,40^ and mucus^27^), based on their traits (e.g. predatory^48^, larger^30^, group living and/or sedentary^31^) or abundance (more abundant reef species visit cleaners frequently and show reduced ectoparasite loads^39^), influencing their value to a cleaner. This explains why here, the functional identity of different clients predicted their need to seek out cleaning services. However, this result was dynamic across time, and thus we suggest that the environmental context acting upon a partners’ traits, at a specific timepoint, may also indirectly influence the behaviours of partners to one another. For cleaning, the ectoparasitic diversity on clients for example, will fluctuate across time and space, since the general community of parasites within an environment can depend heavily on external conditions (e.g. biotic factors: host to parasite ratio and, host phenotypic and genetic diversity within an environment^29,30,40,49,50^)—dynamically influencing the need for clients to seek out cleaning. Context-dependency can hence regulate mutualistic outcomes both directly and indirectly.

For cleaners like the sharknose goby (*Elacatinus evelynae*) observed in the current study, who gain all their nutrition and energy from client derived material, tempo-spatial fluctuations in certain resources is not optimal. Higher energy gains can be obtained through consuming higher quality foods, feeding for longer and increasing diet breadth^51^ and indeed here cleaning frequency increased with the diversity of clients cleaned, and rarer clients in the wider environment, were also cleaned for longer—cleaners may be capitalising on less frequent visitors^35^. However, factors relating to client assemblage (number species cleaned and locally available, and local client abundance) were the consistent predictors of cleaning frequency, irrespective of changing external contexts. This suggests that rather than consistently adopting a cleaning strategy that produces the highest energy gains from each interaction, cleaners instead may be simply capitalising on the large diversity and abundance of client species available to them, gaining their optimal nutrition/energy through interacting with different context-dependent client types. Interacting with multiple partners (through increased abundance and/or richness) could provide a cumulative return to the cleaner and produce more consistency in returns across time^52^ since individual clients will provide maximal food rewards under different environmental conditions. In addition, different clients may produce complimentary effects over time, since a cleaner’s nutritional needs will change across its’ ontogeny (sharknose goby cleaners are thought to be relatively short lived; mean age < 50 days^53^) and with the temporal sequence of cleaner-client interactions throughout the day^54^. This hypothesis, that cleaners are adopting a diversified bet-hedging strategy^52^, is further supported by the lack of consistent predictors found here for interaction durations: instead these measures may be driven by unquantified features of the partner, such as physiological state and metabolism^51^, which could influence the amount of investment by an individual in each interaction. Across time, cleaning can hence be maintained as a stable food source through choice options, irrespective of parasite induced shifts in food availability and diversity within and across client species.

This study also highlights for the first time for cleaning interactions, how the presence of third-party species buffers mutualistic patterns (as also shown for plant-pollinator^55,56^, plant-mycorrhizal^7^ and plant-rhizobium^57^ interactions). Within a diverse community, where individual partners vary in quality, a greater sample of the community is more likely to include the most beneficial partners (sampling effect), promoting the occurrence and maintenance of the mutualism^52^. Incorporating spatiotemporal heterogeneity into mutualistic models is thus more important than ever, since it promotes local spatial variations within an environment in both partner and third-party qualities^16,47^. This perhaps explains why we found the local rather than wider environment to be a more important/consistent predictor of interactions and why interactions differed between neighbouring locations. This finding, that context-dependency modulates cleaning patterns at a local, rather than wider scale, has important implications for our highly debated understanding of how mutualisms influence species diversity, and vice versa^8^. With different local conditions promoting differences in mutualistic patterns, a mosaic of asymmetric patterns will occur across the wider environment: an ideal partner in one local environment at a particular timepoint, may not be ideal in another^6^. This tempo-spatial segregation of partners will ultimately promote tempo-spatial niche-partitioning, facilitating the coexistence of species^6,8,52^. Thus, local mutualists not only rely on partner diversity to persist in a heterogenous landscape, but their local persistence and success could also indirectly promote partner diversity within the wider environment. The magnitude of this effect should increase with mutualism dependence and the number of partners involved in the interaction^52^. Especially for cleaners, which rely on their coral head cleaning stations for client visitation, the direct and indirect role of local microhabitat variations in influencing mutualistic interaction patterns must now be considered: certain local microhabitat traits (e.g. complexity) may influence the availability, diversity and distribution of partner species within the local environment^58,59^, constraining or facilitating mutualistic interactions.

Through quantifying both cleaner and client behaviour over 8 years, and identifying the relative importance of multiple contextual factors for mutualistic outcomes^33^, this study hints at how mutualistic cleaning patterns will shift along species diversity gradients. We highlight how partner choice at a local scale consistently regulates the frequency of an iconic mutualistic cleaning interaction. We thus propose that mutualisms as we know them today (especially those which have choice options e.g. cleaner-client and plant-pollination) could be balanced optimally on a diversity regulated parabolic curve. Too much diversity, and the pervasiveness of mutualisms within an environment may decline—if mutualists gain their necessary benefits quickly through only interacting with a number of high-quality partners, the opportunities for low-quality partners to engage in interactions will reduce^52^. Too little diversity, and mutualists may not gain enough benefits across time and space for the interaction to be sustained, reducing the stability and hence prevalence of the mutualism (as already occurring for some pollinators^64^ and ant-plant mutulisms^41,43^). Across our ecosystems, biodiversity is rapidly declining as a result of natural and human-induced disturbances^9^, it is thus not clear how key ecosystem services provided by mutualistic outcomes (e.g. pollination^11^ and parasite control^12^) will function if mutualisms disappear.

## Methods

### Long-term study site

This 8 year long term study took place on the fringing shallow reef (1–2 m water depth) area (70 m × 60 m) of Booby Reef, Man O’ War Bay Tobago (11°19.344′N 060°33.484′W; see Dunkley et al.^20^ for more detailed site description). Sharknose goby (*Elacatinus evelynae*) cleaners show site fidelity to their brain coral cleaning stations^22^, and stations were marked each year and matched between years (total number long-term stations across 8 years = 82, see Table 1 for within year sample sizes). Long-term stations were defined as those which were occupied by a cleaner in at least two different years. The location of each station on the reef was mapped using GPS, and individual stations were located at least 1 m apart from one another. Within each year, not all marked stations were occupied by sharknose gobies (Table 1). Individual sharknose gobies have high turnover rates on their cleaning stations (mean age < 50 days^53^) and thus different individuals will have been observed at the same cleaning stations across years. The number of gobies occupying each station, within years, ranged from one to nine. It is not possible to naturally identify individual gobies in situ, and thus the cleaning behaviour of different individuals will have also been observed at the same station within each year. This study thus represents cleaner-client interaction patterns over the years, irrespective of which cleaning goby individuals are occupying the station.

**Table 1:** Number of occupied sharknose goby (*Elacatinus evelynae*) cleaning stations on Booby Reef, Man O’ War Bay Tobago over 8 years of long-term study.

Year	Number occupied long-term stations	Total number cleaning observations	Mean (± standard error) number of observations per station
2010	15	61	4.07 ± 0.86
2011	32	271	8.47 ± 0.74
2012	31	233	7.52 ± 0.78
2013	21	108	5.14 ± 0.47
2014	24	143	5.96 ± 0.87
2015	22	166	7.55 ± 0.87
2016	60	290	4.83 ± 0.40
2017	59	267	4.53 ± 0.38

Multiple 10 min cleaner-client observations were carried out at each occupied station.

### Quantifying cleaner-client interactions

Cleaning interactions were observed each year using snorkelling over a 2 week (2010–2015; June) or 6 week (2016–2017; May/June/July) period between the hours of 07:30 to 17:00 (total number of observations across years = 1539). Despite differences in the sampling time length across years the number of observations within each year did not differ substantially (Table 1, mean number of observations = 192). The identity (species), duration and frequency of cleaning of, and posing by, client species during each observation was recorded as a measure of cleaner-client behaviour. Posing involves a client presenting their body to the focal cleaner^19^. Where multiple cleaners were observed on one station, a focal individual was randomly selected for each observation (Table 1 for sample size) and was observed for 10 min.

### Defining contextual factors

To identity factors that are important in governing cleaner-client interactions, data were collected on 12 additional variables which represent the categories of partner identity, partner abundance and the presence of third-party species (Table 2). The presence of third-party species was defined here as the species in the community that are external, but available, to the focal mutualism at a specific time point (adapted from Bronstein and Barbosa^32^).

**Table 2:** Detailed descriptions of contextual factors used to predict cleaning and posing behaviours across and within 8 years.

Category	Factor	Definition
Partner identity (PI)	Client functional group	FishBase^60^ was used to record clients as either solitary or gregarious (associate with > 3 individuals) and sedentary or free-ranging
	Client size	Client species assigned fork lengths using^61^. The upper value of the general size range was used. Range 9–150 cm
	Client trophic level	Client species assigned trophic levels using FishBase^60^. Range 2–4.4
Partner abundance (PA)	Client local abundance	Posing frequencies were combined with the frequency of clients swimming by the focal cleaner (within 20 cm)
	Client wider environment abundance	Median per minute values of each client species based on n = 19 (per year) 50 min random swim surveys
	Cleaner local abundance	Number of gobies occupying station for the observation. Range 0–9
	Cleaner wider environment abundance	Mean number of gobies occupying the stations within each year. Range 0.6–1.28
Presence of third-party species (TP)	Number species cleaned	Number of different species observed being cleaned within each observation. Range 0–7
	Number species locally available	Number of different species observed posing at and/or swimming by the cleaning station within each observation. Range 0–14
	Number species in wider environment	Based on fish counts at the start of June and cleaning observations. Range 45–78 spp.
	Client local relative abundance	Relative abundance of clients at the station, based on ‘client local abundance’ and the total local abundance of different species at the station. Range 0–1
	Abundance other cleaner species	Based on fish species counts used to identify ‘client wider environment abundance’. Range 0.72–3.19

Factors are illustrated in Fig. 2.

#### Partner identity factors

As sharknose gobies were the focus of behavioural observations, partner identity related to the client. Each client species that was observed posing and/or being cleaned, was assigned values for their body length (Table 2, upper value of the general size range of species observed by divers obtained from Humann and Deloach^60^: it was not possible to gain a standardised measure from FishBase^61^ due to inconsistencies in which length is reported and yearly/location differences in body size) and trophic level. Clients were also grouped based on their sociality (gregarious versus solitary) and mobility (free-ranging versus sedentary) behaviour (Table 2)^61^. This meant that three contextual factors (functional group, trophic level and size) were used to represent client partner identity (Fig. 2).

#### Partner abundance factors

Abundance surveys and client data from behavioural observations were used to quantify partner abundance, which was represented by four contextual factors (Fig. 2, Table 2). The abundance’s of client species in the wider reef environment were recorded each year at the start of June using 50 min random swim surveys (n = 19 per year), and the median numbers of fish per minute was calculated for each species which reduced the skew effect of species patchiness (e.g. shoaling behaviours). Client local abundance was quantified by recording the frequency of clients swimming by the focal cleaner (within 20 cm) during the 10 min cleaning observations. For analyses investigating cleaning patterns, the clients’ local abundance was calculated by combining posing and swimming frequencies at the station, whilst for models relating to posing, only the client’s swimming frequency was used. Cleaner wider and local abundance were quantified based on the number of sharknose gobies occupying each cleaning station during randomly timed multiple presence-absence surveys, which occurred daily.

#### Presence of third-party species factors

Five factors were used to represent the presence of third-party species (Fig. 2, Table 2). The number of species cleaned and locally available for cleaning was quantified for each 10 min observation: local availability represented the number of client species posing at and/or swimming by (within 20 cm of) the cleaner (only swimming by for posing analyses). The clients’ local relative abundance at the station represented the percentage of times the species was observed posing and/or swimming by the station whilst accounting for the number of times other species interacted with the station (Table 2). The number of client species in the wider environment was quantified by combining the diversity of species observed on abundance swims and during behavioural observations. Finally, swim surveys also provided information on the abundance of other cleaner species that were present on the reef every year (Table 2, three additional part-time cleaner species present: *Bodianus rufus*, *Pomacanthus paru* and *Thalassoma bifasciatum*).

### Data analysis

To investigate how cleaner-client interactions vary tempo-spatially, and to determine the effect of 12 context-dependent factors on cleaning and posing frequency/duration, we used Generalised Linear Mixed Models (GLMMs) which were run using lme4^62^ in R, version 3.4.3^63^. Cleaning and posing frequency data represent the summed interaction frequency for each client species within each observation, whilst cleaning/posing duration data represented each single individual cleaning/posing event and its respective interaction length. The total time for each focal observation accounted for the amount of time a cleaner was out of view, and thus varied across observations. Cleaning and posing frequencies and durations were therefore weighted by observation length.

To investigate patterns in cleaning and posing frequencies a binomial model with a probit link was specified. For investigating patterns in cleaning and posing durations, which represented the proportion of time each individual client spent interacting with a cleaner, a logit function was applied, and the absolute values were taken, before specifying models with Gaussian families and log links. These model structures were used for all analyses. All models contained the random effect of station number to account for multiple observations within and across years at each station. Each observation across the 8 year dataset (n = 1539), was assigned a unique observation ID number and for all duration models, where multiple cleaning/posing interactions were observed within an observation, observation ID was specified as a random effect. Observer type, which classified observers as those which collected data within a single year (n = 5) or across multiple years (n = 7), was also included as a fixed effect in all models. If ‘observer type’ significantly predicated a response value in any of the models, we checked whether its inclusion or exclusion influenced the significance of the other significant fixed effects—in all cases (n = 3) the inclusion of ‘observer type’ had no effect on result significance.

To find best fitting models, all models were refined by stepwise deletion. Model structures were also checked using a forward stepwise approach, to ensure that final models were not simply influenced by including a large number of predictors^64^. Model assumptions and fits were assessed using residual plots^65^. Binomial models were checked for overdispersion by calculating the ratio between the sum of the squared Pearson residuals and the residual degrees of freedom (value > 2 indicates excessive overdispersion^66^). To facilitate model convergence (and to gain standardised β values for comparison), all continuous predictors were scaled and centered around zero, and models were run without using the Laplace approximation (nAGQ = 0). The significance of fixed effects was assessed using likelihood ratio tests, whilst their importance was defined by observing the proportion change in _adjusted_R^2^ that each predictor produces when it is added last to the final model or through comparing β values. Tukey’s tests were used for any *post-hoc* analysis.

To test the hypothesis that cleaner-client interactions are tempo-spatially dynamic across 8 years, time of day and year were specified as fixed effects in four GLMM models (response variables: cleaning/posing frequency, cleaning/posing duration). Time of day was also nested within each year to determine whether time of day predicted cleaning and posing behaviours differently within each year. To determine whether different stations differed in their observed cleaning and posing behaviours, best fitting models with and without the random effect of station ID were compared using likelihood ratio tests. For spatial analyses, predicted values for cleaning and posing frequencies and durations using GLMM response results, were calculated from final temporal models containing only significant predictors. Using these predicted values, it was then determined whether the mean values for each station (and separately their relative standard error) were spatially autocorrelated using station GPS positions and Mantel’s tests. Finally, to determine whether cleaning stations that were clustered with others differed in the cleaning and posing behaviours to those that were more isolated, the presence of significant correlations between mean predicted values for each station and behaviour and their degree of aggregation were checked for. Aggregation scores were based on a PC1 value calculated from the nearest neighbour distance, the number of stations within 3 m (based on observed swimming distances of cleaners) and the number of stations within 5 m (based on maximum distance a cleaner was observed swimming from its station across the whole 8 year study).

To investigate which contextual factors are the most important for predicting mutualistic outcomes within and across years, all 12 contextual factors (Table 2) were specified as fixed effects in four models (response variables: cleaning/posing frequency, cleaning/posing duration). Models were checked for multicollinearity using the variance inflation factor. Models were first refined based on data across all 8 years of study. The importance of each significant predictor for cleaning and posing behaviours was assessed using changes in _adj_R^2^ values when the term was added last to the model. To determine whether significant predictors of cleaning/posing frequency/duration were also significant within years, each significant predictor was nested within the categorical factor ‘Year’ and z-values were used to assess within year significance. When a categorical factor was significant across years, its within year significance was assessed by sub-setting the data by year and calculating likelihood ratio values.

To determine how consistent contextual factors are in predicting cleaning and posing frequencies and durations and to strengthen the within-year analysis, models were re-run 1000 times with sub-sampled long-term data (n = data from 192 observations). The amount of sub-sampled data, chosen at random for each model simulation, was based on the mean number of 10 min observations carried out within each year. This process was run for the four cleaner-client behaviours (cleaning and posing frequencies and durations). From simulated models likelihood ratio test results for the significance of each contextual factor in predicting cleaner-client behaviours were extracted, along with β coefficient values. The proportion of times that the factor significantly predicted the response variable determined which contextual factors were consistent predictors of cleaner-client behaviour. Contextual factors which significantly predicted cleaner-client behaviours in 95% of times (α = 0.05) were considered consistent predictors, whilst those with *p* > 0.05 were defined as dynamic predictors of cleaning/posing. The β coefficient values shows the effect direction of each contextual factor (except for the categorical ‘client functional group’: this was not an issue as ‘client functional group’ was never a consistent predictor of cleaning or posing behaviours). To determine which consistent contextual predictors were more important in predicting cleaner/client behaviours, each consistent predictor within each model was ranked based on their absolute β value, with higher β values indicating a more important contextual factor (factors were all zero-centred for comparison). P-values were subsequently calculated representing the proportion of times (from 1000 models) that each contextual factor was ranked as most important: if *p* < 0.05 it suggested that the contextual factor was not the most important, relative to others, predictor of cleaning/posing behaviour.

## Supplementary information

Supplementary information.

## Data Availability

Data and code used for this manuscript are available from the corresponding author on reasonable request.
