# Supplementary material for: Consistency in mutualism relies on local, rather than wider community biodiversity
Source: Sci Rep. 2020 Dec 4;10:21255. doi: 10.1038/s41598-020-78318-x (PMC7718221; doi:10.1038/s41598-020-78318-x)
Supplement: Supplementary file 1 — Supplementary information. [file 41598_2020_78318_MOESM1_ESM.pdf]

Consistency in mutualism relies on local, rather than wider community biodiversity

Katie Dunkley[1,2*], Jo Cable[1#], Sarah E. Perkins[1#]

[1] School of Biosciences, Cardiff University, Cardiff, CF10 3AX, UK

[2] Department of Zoology, University of Cambridge, CB2 3EJ, UK

* Corresponding author: kd482@cam.ac.uk

* Corresponding author ORCID ID: https://orcid.org/0000-0002-6032-6577

# Joint Senior Authors

SUPPLEMENTARY INFORMATION

Supplementary Table 1: GLMM model outputs determining whether cleaning and posing behaviours differed tempo-spatially.

	Cleaning frequency	Cleaning duration	Posing frequency	Posing duration
Year	χ^2_7 = 34.42, p < 0.001	χ^2_7 = 11.17, p = 0.132	χ^2_7 = 78.51, p < 0.001	χ^2_7 = 22.89, p = 0.002
Year pattern	*See Supplementary Figure 1*			
Time of day	χ^2_1 = 0.07, p = 0.790	χ^2_1 = 0.05, p = 0.816	χ^2_1 = 0.96, p = 0.327	χ^2_1 = -0.018, p = 1.00
Station differences?	χ^2_1 = 22.65, p < 0.001	χ^2_1 = 25.09, p < 0.001	χ^2_1 = 25.09, p < 0.001	χ^2_1 = 0.37, p = 0.543
Spatial patterning?	r = -0.030, p = 0.801	r = 0.051, p = 0.151	r = -0.005, p = 0.492	r = 0.02, p = 0.262
Degree of aggregation	r = -0.08, p = 0.501	r = -0.06, p = 0.610	r = 0.04, p = 0.697	r = 0.01, p = 0.943

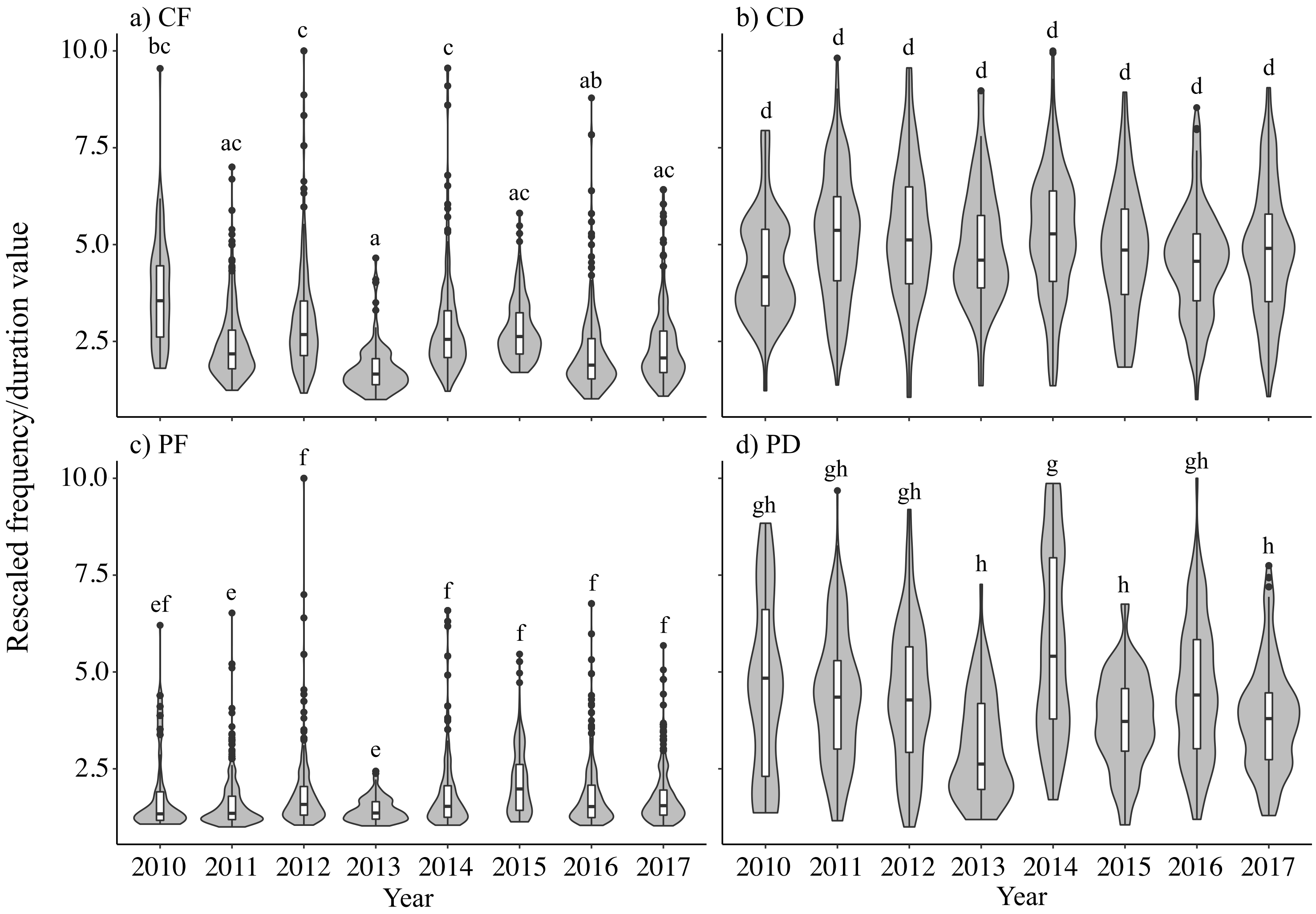

Supplementary Figure 1: Sharknose goby (*Elacatinus evelynae*) cleaning frequencies (a – CF) and durations (b – CD) and their clients' posing frequencies (c – PF) and durations (d – PD) across 8 years. Predicted values from GLMMs are plotted, with the violin plots showing the distribution

of cleaning/posing frequency/duration values observed within each year (2010 – 2017), whilst box plots show median and inter-quartile ranges of predicted values. Letters indicate Tukey *post-hoc* groupings and identify significant differences between years. Predicted values have been rescaled from 1 – 10 to enhance figure clarity, with increased values representing increased frequencies/durations.

Supplementary Table 2: Significant GLMM predictors of sharknose goby (*Elacatinus evelynae*) cleaning frequencies and durations and their clients posing frequencies and durations across 8 years. Contextual factors are nested with the categories partner identity (PI), partner abundance (PA) and the presence of third-party species (TP).

Cat	Factor	Cleaning		Posing	
		Frequency	Duration	Frequency	Duration
PI	Client functional group	$\chi^2_3 = 148.09$, $p < 0.001$	$\chi^2_3 = 13.77$, $p = 0.003$	$\chi^2_3 = 241.88$, $p < 0.001$	$\chi^2_3 = 50.00$, $p < 0.001$
	Client trophic level	$\chi^2_1 = 278.11$, $p < 0.001$	-	$\chi^2_1 = 211.05$, $p < 0.001$	$\chi^2_1 = 14.10$, $p < 0.001$
	Client size	$\chi^2_1 = 8.18$, $p = 0.004$	$\chi^2_1 = 21.44$, $p < 0.001$	$\chi^2_1 = 13.46$, $p < 0.001$	-
PA	Client local abundance	$\chi^2_1 = 214.00$, $p < 0.001$	-	$\chi^2_1 = 99.27$, $p < 0.001$	-
	Client wider abundance	-	$\chi^2_1 = 35.12$, $p < 0.001$	$\chi^2_1 = 6.02$, $p = 0.014$	-
	Cleaner local abundance	$\chi^2_1 = 4.38$, $p = 0.036$	-	$\chi^2_1 = 6.58$, $p = 0.010$	-

	Cleaner wider abundance	-	-	$\chi^2_1 = 10.50$, $p = 0.001$	-
TP	Number species cleaned	$\chi^2_1 = 585.22$, $p < 0.001$	-	$\chi^2_1 = 36.46$, $p < 0.001$	-
	Number species locally available	$\chi^2_1 = 271.36$, $p < 0.001$	-	-	-
	Client local relative abundance	$\chi^2_1 = 21.01$, $p < 0.001$	-	$\chi^2_1 = 106.66$, $p < 0.001$	-
	Number species in wider environment	$\chi^2_1 = 9.53$, $p = 0.002$	$\chi^2_1 = 4.36$, $p = 0.037$	-	-
	Abundance other cleaner species	$\chi^2_1 = 10.58$, $p = 0.001$	-	$\chi^2_1 = 52.13$, $p < 0.001$	-

Supplementary Table 3: Within year significance of each of the significant long-term predictors of cleaning frequencies (a), durations (b) and posing frequencies (c) and durations (d). Significant years are shaded in grey. Some variables were quantified once per year and thus did not vary within years (indicated by '-').

(a) Cleaning frequency

	2010	2011	2012	2013	2014	2015	2016	2017
Client functional group	$\chi^2_3 = 4.51$, $p = 0.212$	$\chi^2_3 = 3.48$, $p = 0.323$	$\chi^2_3 = 20.20$, $p < 0.001$	$\chi^2_3 = 3.45$, $p = 0.327$	$\chi^2_3 = 58.09$, $p < 0.001$	$\chi^2_3 = 4.98$, $p = 0.174$	$\chi^2_3 = 60.51$, $p < 0.001$	$\chi^2_3 = 54.74$, $p < 0.001$

Client trophic level	z = -2.10, p = 0.036	z = -4.83, p < 0.001	z = -6.26, p < 0.001	z = -4.53, p < 0.001	z = -6.62, p < 0.001	z = -4.34, p < 0.001	z = -6.92, p < 0.001	z = -5.70, p < 0.001
Client size	z = -0.98, p = 0.327	z = -0.92, p = 0.356	z = -0.89, p = 0.373	z = 0.26, p = 0.798	z = 3.02, p = 0.002	z = 0.42, p = 0.674	z =1.39, p = 0.164	z = 6.37, p < 0.001
Client local abundance	z = 3.54, p < 0.001	z = 2.16, p = 0.031	z = 7.56, p < 0.001	z = 0.56, p = 0.577	z = 7.16, p < 0.001	z = 4.04, p < 0.001	z = 7.39, p < 0.001	z = 1.76, p = 0.079
Client local abundance	z = -1.15, p = 0.249	z = -1.34, p = 0.179	z = 0.69, p = 0.488	z = -0.64, p = 0.523	z = -0.73, p = 0.463	z = 0.99, p = 0.323	z = -1.10, p = 0.270	z = -2.28, p = 0.023
Number species cleaned	z = 3.16, p = 0.002	z = 6.65, p < 0.001	z = 9.69, p < 0.001	z = 7.36, p < 0.001	z = 8.75, p < 0.001	z = 7.66, p < 0.001	z = 13.56, p < 0.001	z = 12.63, p < 0.001
Number species locally available	z = -3.20, p = 0.001	z = -5.57, p < 0.001	z = -5.30, p < 0.001	z = -3.04, p = 0.002	z = -5.04, p < 0.001	z = -5.46, p < 0.001	z = -7.39, p < 0.001	z = -3.53, p < 0.001
Client local relative abundance	z = -4.63, p < 0.001	z = -5.11, p < 0.001	z = -2.38, p = 0.017	z = -0.68, p = 0.498	z = 0.93, p = 0.354	z = -1.96, p = 0.050	z = -0.91, p = 0.362	z = 2.22, p = 0.027
Number species in wider environment				-				
Abundance other cleaner species				-				

(b) Cleaning duration

	2010	2011	2012	2013	2014	2015	2016	2017
Client functional group	$\chi^2_3 = 11.97$, $p = 0.007$	$\chi^2_3 = 21.68$, $p < 0.001$	$\chi^2_3 = 3.44$, $p = 0.328$	$\chi^2_3 = 4.92$, $p = 0.177$	$\chi^2_3 = 11.55$, $p = 0.009$	$\chi^2_3 = 5.42$, $p = 0.144$	$\chi^2_3 = 1.85$, $p = 0.605$	$\chi^2_3 = 1.94$, $p = 0.585$
Client size	$t = 0.90$, $p = 0.365$	$t = 0.85$, $p = 0.395$	$t = 4.57$, $p < 0.001$	$t = 2.17$, $p = 0.030$	$t = 0.58$, $p = 0.562$	$t = 1.47$, $p = 0.141$	$t = 1.83$, $p = 0.067$	$t = 0.56$, $p = 0.578$
Client wider abundance	$t = 0.40$, $p = 0.690$	$t = -4.25$, $p < 0.001$	$t = -2.40$, $p = 0.017$	$t = -1.44$, $p = 0.149$	$t = -2.18$, $p = 0.029$	$t = -0.98$, $p = 0.326$	$t = -0.30$, $p = 0.763$	$t = -0.68$, $p = 0.495$
Number species in wider environment	-							

(c) Posing frequency

	2010	2011	2012	2013	2014	2015	2016	2017
Client functional group	$\chi^2_3 = 5.62$, $p = 0.131$	$\chi^2_3 = 19.72$, $p < 0.001$	$\chi^2_3 = 15.61$, $p = 0.001$	$\chi^2_3 = 15.48$, $p = 0.001$	$\chi^2_3 = 128.13$, $p < 0.001$	$\chi^2_3 = 6.37$, $p = 0.095$	$\chi^2_3 = 35.02$, $p < 0.001$	$\chi^2_3 = 39.52$, $p < 0.001$
Client trophic level	$z = -2.16$, $p = 0.031$	$z = -4.03$, $p < 0.001$	$z = -1.85$, $p = 0.065$	$z = -4.20$, $p < 0.001$	$z = -6.69$, $p < 0.001$	$z = -4.09$, $p < 0.001$	$z = -5.75$, $p < 0.001$	$z = -5.55$, $p < 0.001$
Client size	$z = -2.04$, $p = 0.041$	$z = -3.13$, $p = 0.002$	$z = -1.60$, $p = 0.111$	$z = 0.18$, $p = 0.857$	$z = 0.17$, $p = 0.863$	$z = -2.16$, $p = 0.031$	$z = -2.23$, $p = 0.026$	$z = 0.81$, $p = 0.418$

Client local abundance	z = 1.51, p = 0.130	z = -1.89, p = 0.059	z = 0.75, p = 0.454	z = 0.73, p = 0.463	z = 1.96, p = 0.050	z = -2.77, p = 0.006	z = 4.54, p < 0.001	z = 5.54, p < 0.001
Client wider abundance	z = -1.39, p = 0.165	z = 3.48, p < 0.001	z = 3.59, p < 0.001	z = 0.74, p = 0.462	z = -0.66, p = 0.511	z = -0.25, p = 0.801	z = 3.79, p < 0.001	z = 1.51, p = 0.132
Cleaner local abundance	z = 0.22, p = 0.829	z = 0.10, p = 0.917	z = 0.29, p = 0.769	z = -1.25, p = 0.211	z = -1.04, p = 0.299	z = 0.18, p = 0.860	z = -2.96, p = 0.003	z = -1.63, p = 0.103
Cleaner wider abundance	-							
Number species cleaned	z = 0.56, p = 0.579	z = -0.63, p = 0.528	z = 2.48, p = 0.013	z = 2.66, p = 0.008	z = 2.74, p = 0.006	z = 0.64, p = 0.519	z = 3.37, p < 0.001	z = 2.73, p = 0.006
Client local relative abundance	z = -2.19, p = 0.029	z = -2.38, p = 0.017	z = -2.65, p = 0.008	z = -3.11, p = 0.002	z = -6.33, p < 0.001	z = -2.19, p = 0.029	z = -1.37, p = 0.172	z = -3.54, p < 0.001
Abundance other cleaner species	-							

(d) Posing duration

	2010	2011	2012	2013	2014	2015	2016	2017
Client functional group	χ^2_3 = 2.02, p = 0.364	χ^2_3 = 18.23, p < 0.001	χ^2_3 = 3.86, p = 0.277	χ^2_3 = 11.93, p = 0.008	χ^2_3 = 26.41, p < 0.001	χ^2_3 = 3.80, p = 0.284	χ^2_3 = 0.87, p = 0.832	χ^2_3 = 6.10, p = 0.107
Client trophic level	t = 0.22, p = 0.824	t = 1.36, p = 0.175	t = 2.76, p = 0.006	t = 1.05, p = 0.293	t = 1.51, p = 0.131	t = 1.54, p = 0.124	t = 0.25, p = 0.803	t = 0.09, p = 0.926
